# Measuring the Electro-Optical Kerr Effect Against the Background of Electro-Absorption Modulation in Liquids

**DOI:** 10.3390/ma17215346

**Published:** 2024-10-31

**Authors:** Rafał Ledzion, Marek Izdebski, Anita Rambo

**Affiliations:** Institute of Physics, Lodz University of Technology, Wólczańska 217/221, 93-005 Łódź, Poland; rafal.ledzion@p.lodz.pl (R.L.);

**Keywords:** electro-optical Kerr effect, polarimetric technique, electro-absorption, orientational ordering

## Abstract

A new approach to the dynamic polarimetric method is proposed, which allows for the decoupling of electro-optical Kerr effect measurements from the electro-absorption effect in partially transparent liquids. The method is illustrated by using the results of engine oil measurements as a function of temperature and modulating field frequency. It was shown that the birefringence induced in the sample, the modulation of the ordinary wave transmission, and the modulation of the extraordinary wave transmission in the sample can be shifted in phase with respect to the square of the applied alternating modulating field. Each of these three phase shifts can depend differently on the temperature and frequency. Neglecting the influence of electro-absorption on electro-optical measurements in liquids or considering electro-absorption as an effect correlated in phase with induced birefringence may lead to significant measurement errors. This indicates that the Kerr constant and the electro-absorption coefficients for an alternating electric field should be considered as complex quantities instead of real values, as they have been traditionally. The proposed approach fills an important gap in measurement techniques described in the literature, which may provide erroneous results for measurements of the Kerr constant in partially transparent liquids including many industrially important liquids.

## 1. Introduction

The electro-optical Kerr effect is a linear birefringence Δ*n* induced in a liquid or gas sample under the influence of a DC or a slowly varying electric field *E* applied perpendicularly to the light beam:

(1) $$\Delta n=n_{e}-n_{o}=\lambda KE^{2},$$

where *n*_e_ and *n*_o_ are the refractive indices of the extraordinary and ordinary waves, respectively, *λ* is the is the wavelength of the light, and *K* is the Kerr constant. Traditionally, the *K* constant is considered as a real quantity, which takes positive or negative values depending on the substance under consideration. Reliable measurements of the Kerr effect are essential, because the effect is used in optical switches [1], voltmeters for remote measurement of high voltages [2], electric field mapping [3], and assessing the suitability of various oils for use in transformers [4], among other applications. Experimental data on the Kerr effect are also necessary to select the optimal immersion liquids for electro-optical measurements in solid crystals. The immersion liquids should not significantly disturb the measurements when subjected to the electric field around the electrodes on the crystal, which means that the *K* constant should take the smallest possible values of the order of 10^−15^ m V^−2^ as in insulation mineral oils and methyl silicone oils measured in AC fields [5,6]. Moreover, measurements of the Kerr constant seem to be a promising, cost-effective, and rapid method for the qualitative assessment of various industrially important liquids. It has been shown that aging processes significantly affect the Kerr constants of olive oil [7], transformer oil [8], and medical castor oil [9] measured in an alternating electric field. Although the values of the *K* constant for olive oil are of the order of 10^−9^ m V^−2^, while for castor oil it is only 10^−14^ m V^−2^, a similar change from an increasing frequency dependence of the *K* constant for fresh oil to a decreasing dependence for aged oil was observed for both liquids. In the case of transformer oil, the aging process caused an increase in the *K* constant from 1.8 × 10^−15^ to 3.5 × 10^−15^ m V^−2^ at 23 °C and a frequency of 417 Hz. Because each assessment technique has its limitations, new methods and combinations of methods are still needed to improve the detection limit (see, e.g., a review of techniques for detecting olive oil adulteration in Ref. [10]).

Reliable measurements of the Kerr effect are also important for basic theoretical research. The theory developed by Buckingham and Raab in 1957 [11], as an extension of the Langevin–Born theory, is still the most general known theory of the electro-optical Kerr effect. The main prediction of this theory is the temperature dependence of the molecular Kerr constant in the form $K_{m}=A+B/T+C/T^{2}$, where *A*, *B*, *C* are constants characteristic of the substance and the $C/T^{2}$ term disappears in the case of molecules without a permanent dipole moment. Unfortunately, this theory describes the Kerr effect only in a static electric field, which is insufficient to interpret much experimental data. Independently of the Buckingham and Raab model, a theory of the dynamic Kerr effect was developed based on the forced rotational diffusion model [12]. Recently, an analytical solution for the dependence of electric-field-induced birefringence on the frequency of a sinusoidally modulating field was found in Ref. [13]. While appreciating this advance, we must note that it is still not a general solution. In particular, it is unclear how to combine predictions of frequency dependence with temperature dependence. Moreover, the theoretical frequency dependence of the induced birefringence tends to zero as the expression $a_{1}/\omega +a_{2}/\omega^{2}$, which is inconsistent with the experimental data presented in Section 4 of the current paper.

A review of the literature showed that all classical techniques for measuring the Kerr constant are based on the assumption of complete sample transparency [14,15]. This assumption greatly simplifies the measurement procedure by neglecting the dichroism induced in the sample by an applied electric field. However, in our opinion, this assumption seems questionable for some liquids including many vegetable, mineral, and synthetic oils of industrial importance. In these liquids, we observed modulation of the transmission coefficients of the ordinary and extraordinary waves in the liquid at the second harmonic of the applied alternating field as well as field-induced changes in the DC components of these coefficients. Consequently, the oversimplified model of the liquid sample may become the main source of measurement error, and estimating measurement uncertainty based on the accuracy of the equipment used may no longer be reliable. This problem also concerned all oils studied in [7,8,9], which were only partially transparent and their transparency changed during aging. Therefore, in our opinion, it is not clear whether the previously observed changes in the properties of various oils were correctly interpreted as changes in the *K* constant.

Here, we present a general measurement method that allows for decoupling of the electro-optical Kerr effect from the contribution made by all manifestations of electro-absorption in a sinusoidally alternating electric field. Such a generalized measurement method is necessary for both industrial applications and for the reliable verification of theoretical models. The method presented here is an extension of our previous work [16], in which a method was developed for measuring the quadratic electro-optic effect in solid crystals against the background of the linear effect and natural dichroism. However, field-induced dichroism has not been previously considered.

## 2. Materials and Methods

### 2.1. The Idea of the Measurement Method

Let us consider a Sénarmont-type system consisting of an ideal linear polarizer with an azimuth of *α* = ±45°, a liquid sample placed in a uniform electric field that defines a reference azimuth of 0°, an ideal quarter-wave plate with an azimuth of a fast wave *θ* = ±45°, and an ideal linear analyzer with variable azimuth *β*. We assumed that the sample was optically inactive and could become dichroic in an electric field. Using the Jones calculus and the general form of the Jones matrix derived in Ref. [17], it can be found that the transmitted light intensity *I* is given by the formula

(2) $$I/I_{p}=\frac{1}{4}\left(S_{e}^{2}+S_{r}^{2}\right)T_{\mathrm{qa}}^{2}+\frac{1}{2}pS_{e}S_{r}T_{\mathrm{qa}}^{2}\sin\left(2\beta -q\Gamma \right),$$

where *I*_p_ is the light intensity behind the polarizer, *S*_e_ and *S*_r_ are the amplitude transmission coefficients of the extraordinary and ordinary waves in the sample, respectively, *T*_qa_ is the total amplitude transmission coefficient through the non-dichroic quarter-wave plate and the analyzer, Γ is the phase difference between the extraordinary and ordinary waves in the sample, and *p* and *q* take values ±1 depending on the azimuths of the polarizer and quarter-wave plate: *p* = sgn(*α*), *q* = sgn(*θ*).

We did not expect any birefringence in an isotropic liquid when the field was turned off. However, when liquid is placed in a glass or quartz spectrophotometric cuvette, the optical windows of the cuvette may introduce a birefringence of up to several degrees. Therefore, we describe the total phase difference in the sample as the sum of field-free birefringence and birefringence resulting from the Kerr effect as

(3) $$\Gamma =\Gamma_{0}+2\pi \mathrm{LK}E^{2},$$

where *L* is the length of the light path through the sample. Let us consider a sinusoidally varying field:

(4) $$E=E_{0}\sin\left(\omega t\right).$$

Substituting Formula (4) into (3) yields the total phase difference as the sum of the DC component and the AC component with a frequency of 2*ω*:

(5) $$\Gamma =\Gamma_{\mathrm{DC}}+\Gamma_{2\omega }=\Gamma_{0}+\pi \mathrm{LK}E_{0}^{2}-\pi \mathrm{LK}E_{0}^{2}\cos\left(2\omega t\right).$$

The definition of the Kerr constant (1), and consequently Formula (5), ignores the dynamics of the Kerr effect, which leads to a fixed phase of the Γ_2*ω*_ component that can only be equal or opposite to the phase of *E*^2^. However, this result is far from corresponding to our experimental data, as presented in Section 4. It was also inconsistent with the recent theoretical results derived in [13] using the forced rotational diffusion model, where a linear combination of contributions proportional to sin(2*ωt*) and cos(2*ωt*) was obtained. Moreover, the middle term $\pi \mathrm{LK}E_{0}^{2}$ in Formula (5) refers to the static Kerr effect, which appears here as a result of field rectification in the expression $E^{2}\sim \sin^{2}\left(\omega t\right)$, whereas the last term describes the dynamic effect. Because the constants for these two manifestations of the effect can be very different, we used the two symbols $K_{0}$ and *K*, with $K_{0}=\underset{\omega \to 0}{\lim}K(\omega )$. Taking the above into account, we can rewrite Equation (5) in a more general form:

(6) $$\Gamma =\Gamma_{\mathrm{DC}}+\Gamma_{2\omega }=\Gamma_{0}+\pi LK_{0}E_{0}^{2}-\pi L\left|K\right|E_{0}^{2}\cos\left(2\omega t+\varphi_{K}\right),$$

where distinguishing between positive and negative values of *K* is no longer necessary because we allowed for any values of *φ*_K_.

In our analysis, we also allowed for field-induced changes in the transmission coefficients *S*_e_ and *S*_r_ that manifested themselves as changes in the DC components *S*_e0_ and *S*_r0_ as well as modulation at the second harmonic of the modulating field:

(7) $$S_{e}=S_{e0}+A_{e}E_{0}^{2}\cos\left(2\omega t+\varphi_{e}\right),$$

(8) $$S_{r}=S_{r0}+A_{r}E_{0}^{2}\cos\left(2\omega t+\varphi_{r}\right),$$

where we did not impose any relations between the coefficients *S*_e0_ and *S*_r0_, the coefficients *A*_e_ and *A*_r_ or between the phases *φ*_e_, *φ*_r_, and *φ*_K_. After substituting Equations (6)–(8) into (2), we obtained an expression that was difficult to analyze in the general case. However, because the modulation of the phase difference is typically small (|Γ_2*ω*_| ≪ 1), we can use the approximations cos(Γ_2_*_ω_*) ≈ 1 and sin(Γ_2_*_ω_*) ≈ Γ_2ω_. We can also neglect all products of small terms at frequency 2*ω*, and consequently express the intensity *I* as the sum of the DC component *I*_0_ and the second harmonic component *I*_2*ω*_:

(9) $$I_{0}/I_{p}T_{\mathrm{qa}}^{2}=\frac{1}{4}S_{e0}^{2}+\frac{1}{4}S_{r0}^{2}+\frac{1}{2}pS_{e0}S_{r0}\sin\left(2\beta -q\Gamma_{\mathrm{DC}}\right)$$

and

(10) $$\begin{array}{ll} I_{2\omega }/I_{p}T_{\mathrm{qa}}^{2} & =\frac{1}{2}S_{e0}A_{e}E_{0}^{2}\cos\left(2\omega t+\varphi_{e}\right)+\frac{1}{2}S_{r0}A_{r}E_{0}^{2}\cos\left(2\omega t+\varphi_{r}\right)\\ & +\frac{1}{2}\mathrm{pq}S_{e0}S_{r0}\cos\left(2\beta -q\Gamma_{\mathrm{DC}}\right)\pi L\left|K\right|E_{0}^{2}\cos\left(2\omega t+\varphi_{K}\right)\\ & +\frac{1}{2}p\left[S_{e0}A_{r}\cos\left(2\omega t+\varphi_{r}\right)+S_{r0}A_{e}\cos\left(2\omega t+\varphi_{e}\right)\right]E_{0}^{2}\sin\left(2\beta -q\Gamma_{\mathrm{DC}}\right) \end{array}.$$

The transmitted light is measured with a photodetector that generates a voltage *U* proportional to the light intensity *I*. The DC component *U*_0_ of the voltage *U* can be measured using a DC voltmeter (e.g., in this work, Keithley multimeter, model 2000, USA). The second harmonic component can be detected using a lock-in amplifier (e.g. EG&G Instruments, model 7265, Germany), which allows for readings of the RMS voltage *U*_2*ω*_ and the phase *φ*_2*ω*_. Following a common convention used in lock-in amplifiers, we assumed that the values of *U*_2*ω*_ are non-negative, the phase *φ*_2*ω*_ ∈ (−180°; +180°], and the instantaneous voltage is given by

(11) $$U(t)=U_{0}+\sqrt{2}U_{2\omega }\sin\left(2\omega t+\varphi_{2\omega }\right).$$

Let us also define a time-invariant complex RMS voltage $\underline{U_{2\omega }}$ representing the voltage value and the phase angle of the second harmonic component, and an analogous complex light intensity $\underline{I_{2\omega }}$:

(12) $$\underline{U_{2\omega }}=U_{2\omega }\left(\cos\varphi_{2\omega }+i\sin\varphi_{2\omega }\right),$$

(13) $$\underline{I_{2\omega }}=\left|\underline{I_{2\omega }}\right|\left(\cos\varphi_{2\omega }+i\sin\varphi_{2\omega }\right).$$

Now, we can rewrite Equation (10) in a simpler form:

(14) $$\begin{array}{ll} \underline{I_{2\omega }}/I_{p}T_{\mathrm{qa}}^{2} & =i\left[\frac{1}{2}S_{e0}\underline{A_{e}}+\frac{1}{2}S_{r0}\underline{A_{r}}\right.\\ & +\frac{1}{2}\mathrm{pq}S_{e0}S_{r0}\cos\left(2\beta -q\Gamma_{\mathrm{DC}}\right)\pi L\underline{K}\\ & \left.+\frac{1}{2}p\left(S_{e0}\underline{A_{r}}+S_{r0}\underline{A_{e}}\right)\sin\left(2\beta -q\Gamma_{\mathrm{DC}}\right)\right]E_{0}^{2}, \end{array}$$

where

(15) $$\underline{K}=\left|K\right|\left(\cos\varphi_{K}+i\sin\varphi_{K}\right),$$

(16) $$\underline{A_{e}}=A_{e}\left(\cos\varphi_{e}+i\sin\varphi_{e}\right),$$

(17) $$\underline{A_{r}}=A_{r}\left(\cos\varphi_{r}+i\sin\varphi_{r}\right).$$

Equation (14) shows that the modulation due to the Kerr effect can be decoupled from the contribution made by changes in the transmission coefficients by calculating the following difference:

(18) $$\underline{I_{2\omega }}\left(\beta =q\Gamma_{\mathrm{DC}}/2\right)/I_{p}-\underline{I_{2\omega }}\left(\beta =q\Gamma_{\mathrm{DC}}/2+\pi /2\right)/I_{p}=\mathrm{ipq}T_{\mathrm{qa}}^{2}S_{e0}S_{r0}\pi L\underline{K}E_{0}^{2},$$

where the term $T_{\mathrm{qa}}^{2}S_{e0}S_{r0}$ is the doubled amplitude of the *I*_0_(*β*)/*I*_p_ dependence given by Equation (9):

(19) $$I_{0,\max}/I_{p}-I_{0,\min}/I_{p}=T_{\mathrm{qa}}^{2}S_{e0}S_{r0}.$$

Due to possible changes in the light intensity emitted by the laser, the intensity *I*_p_ should not be considered constant over the entire measurement procedure. Because a direct measurement of *I*_p_ could disturb the polarization state of the light, we preferred to measure the input light intensity *I*_IN_ using a semi-transparent mirror placed in front of the polarizer, which illuminated an additional photodetector. The voltage *U*_IN_ at the output of this photodetector is proportional to *I*_p_, but when planning an experiment, we must take into account that each rotation of the polarizer may disturb the proportionality.

Conditions *β* = *q*Γ_DC_/2 and *β* = *q*Γ_DC_/2 + *π*/2 in Equation (18) correspond to the maximum-linearity points of the *I* on Γ characteristic, which are commonly used in Sénarmont-type electro-optical modulators. The fact that the maximum-linearity points were difficult to find became the motivation to develop the FDEOM method (the frequency-doubling electro-optic modulation measurement method), employing the minimum-transmission point [14,15]. If the field *E* does not affect the transmissions *S*_e_ and *S*_r_, the minimum-transmission point can be set precisely as the point where *I*_2*ω*_ = 0. Then, the modulator response at the fourth harmonic becomes proportional to the square of the Kerr constant. However, when this assumption is not met, the FDEOM method fails because the *I*_2*ω*_ = 0 condition is not met at the minimum-transmission point and the Kerr effect is not the sole reason for the fourth harmonic response. Moreover, regardless of which fixed operating point is assumed in a given method, many time-consuming adjustments to this point may be necessary due to the static Kerr effect in a rectified alternating field, which may manifest itself much more strongly than the dynamic effect [18]. In our opinion, the solution to these problems is to abandon any fixed absolute positions for the operating points on the *I* on Γ characteristic. A similar concept was proposed in Ref. [19] using a polarimeter with a rotating quarter-wave plate to measure the electro-optical coefficients. However, this method only works with a static electric field. In this paper, we proposed performing a series of *U*_0_, *U*_2*ω*_, and *U*_IN_ measurements at several operating points, only the relative positions of which were controlled precisely by a stepper motor that rotated the analyzer. Using the proportionalities *U* ~ *I* and *U*_IN_ ~ *I*_p_, and following the forms of Formulas (9) and (14), we can express the voltage dependencies on *β* in the form of a Fourier series, in which only selected components can be different from zero:

(20) $$U_{0}/U_{\mathrm{IN}}=a_{00}+a_{02}\cos2\beta +b_{02}\sin2\beta ,$$

(21) $$\underline{U_{2\omega }}/U_{\mathrm{IN}}=\underline{a_{20}}+\underline{a_{22}}\cos2\beta +\underline{b_{22}}\sin2\beta .$$

If we divide the period 2π into 2*n* equal intervals and take measurements at *β_k_* = *kπ*/*n*, the coefficients in Equations (20) and (21) can be found as

(22) $$a_{00}=\frac{1}{2n}\sum_{k=0}^{2n-1}\frac{U_{0}\left(k\pi /n\right)}{U_{\mathrm{IN}}\left(k\pi /n\right)},$$

(23) $$a_{02}=\frac{1}{n}\sum_{k=0}^{2n-1}\frac{U_{0}\left(k\pi /n\right)}{U_{\mathrm{IN}}\left(k\pi /n\right)}\cos\left(2k\pi /n\right),$$

(24) $$b_{02}=\frac{1}{n}\sum_{k=0}^{2n-1}\frac{U_{0}\left(k\pi /n\right)}{U_{\mathrm{IN}}\left(k\pi /n\right)}\sin\left(2k\pi /n\right),$$

(25) $$\underline{a_{20}}=\frac{1}{2n}\sum_{k=0}^{2n-1}\frac{\underline{U_{2\omega }}\left(k\pi /n\right)}{U_{\mathrm{IN}}\left(k\pi /n\right)},$$

(26) $$\underline{a_{22}}=\frac{1}{n}\sum_{k=0}^{2n-1}\frac{\underline{U_{2\omega }}\left(k\pi /n\right)}{U_{\mathrm{IN}}\left(k\pi /n\right)}\cos\left(2k\pi /n\right),$$

(27) $$\underline{b_{22}}=\frac{1}{n}\sum_{k=0}^{2n-1}\frac{\underline{U_{2\omega }}\left(k\pi /n\right)}{U_{\mathrm{IN}}\left(k\pi /n\right)}\sin\left(2k\pi /n\right).$$

The considered period can be shortened to *π*, which reduces the number of necessary measurements to at least four. For the shortened period, the fraction before the Σ symbol in Equations (22)–(27) should be doubled, and the summation should range from *k* = 0 to *n* − 1.

Equations (20) and (21) allow us to find

(28) $$\left(U_{0,\max}-U_{0,\min}\right)/U_{\mathrm{IN}}=2\sqrt{a_{02}^{2}+b_{02}^{2}}$$

and

(29) $$\left[\underline{U_{2\omega }}\left(\beta =q\Gamma_{\mathrm{DC}}/2\right)-\underline{U_{2\omega }}\left(\beta =q\Gamma_{\mathrm{DC}}/2+\pi /2\right)\right]/U_{\mathrm{IN}}=2\left(\underline{a_{22}}\cos\Gamma_{\mathrm{DC}}+q\underline{b_{22}}\sin\Gamma_{\mathrm{DC}}\right),$$

where sin Γ_DC_ and cos Γ_DC_ result from the proportionality of analogous terms containing sin2*β* and cos2*β* in Equations (9) and (20):

(30) $$\sin\Gamma_{\mathrm{DC}}=\frac{-\mathrm{pq}a_{02}}{\sqrt{a_{02}^{2}+b_{02}^{2}}},$$

(31) $$\cos\Gamma_{\mathrm{DC}}=\frac{p\;b_{02}}{\sqrt{a_{02}^{2}+b_{02}^{2}}}.$$

Now, using the proportionality *U* ~ *I*, we can write

(32) $$\sqrt{2}\frac{\underline{U_{2\omega }}\left(\beta =q\Gamma_{\mathrm{DC}}/2\right)-\underline{U_{2\omega }}\left(\beta =q\Gamma_{\mathrm{DC}}/2+\pi /2\right)}{\underline{I_{2\omega }}\left(\beta =q\Gamma_{\mathrm{DC}}/2\right)-\underline{I_{2\omega }}\left(\beta =q\Gamma_{\mathrm{DC}}/2+\pi /2\right)}=\frac{U_{0,\max}-U_{0,\min}}{I_{0,\max}-I_{0,\min}}.$$

After substituting Equations (18), (19), and (28)–(31) into (32), we finally obtain

(33) $$\underline{K}=-i\frac{\sqrt{2}q}{\pi LE_{0}^{2}}\frac{\underline{a_{22}}b_{02}-\underline{b_{22}}a_{02}}{a_{02}^{2}+b_{02}^{2}}.$$

### 2.2. Measurements with a Non-Ideal Quarter-Wave Plate

Let us reconsider the setup described in Section 2.1. Now, we will allow that the actual phase difference *γ* introduced by the quarter-wave plate may differ from the ideal value of 90°, and that the amplitude transmission coefficients *Q*_f_ and *Q*_s_ of the fast and slow waves in the quarter-wave plate may not be equal. Using the Jones calculus again, we obtain

(34) $$\begin{array}{ll} I/I_{p} & =\frac{1}{8}\left(Q_{f}^{2}+Q_{s}^{2}\right)\left(S_{e}^{2}+S_{r}^{2}\right)\\ & +\frac{1}{4}\;\mathrm{pq}\left(Q_{f}^{2}-Q_{s}^{2}\right)S_{e}S_{r}\cos\Gamma \\ & +\frac{1}{8}\;q\sin\left(2\beta \right)\left(Q_{f}^{2}-Q_{s}^{2}\right)\left(S_{e}^{2}+S_{r}^{2}\right)\\ & +\frac{1}{4}\;\cos\left(2\beta \right)Q_{f}Q_{s}\left(S_{e}^{2}-S_{r}^{2}\right)\cos\gamma \\ & +\frac{1}{4}\;p\sin\left(2\beta \right)\left(Q_{f}^{2}+Q_{s}^{2}\right)S_{e}S_{r}\cos\Gamma \\ & -\frac{1}{2}\;\mathrm{pq}\cos\left(2\beta \right)Q_{f}Q_{s}S_{e}S_{r}\sin\gamma \sin\Gamma . \end{array}$$

The terms containing the differences $\left(Q_{f}^{2}-Q_{s}^{2}\right)$ and $\left(S_{e}^{2}-S_{r}^{2}\right)$ make it impossible to decouple the contribution of the Kerr effect from the contribution of electro-absorption based only on the *I*/*I*_p_ on *β* dependence. However, these terms can be eliminated at the cost of doubling the number of necessary measurements using the average light transmission through the modulator in the following two configurations, differing in the settings of the quarter-wave plate and the analyzer:

(35) $$\bar{T}(p,q,\beta )\overset{\mathrm{def}}{=}\frac{1}{2}\;\left[\frac{I(p,q,\beta )}{I_{\mathrm{IN}}(p,q,\beta )}+\frac{I(p,-q,\;90{}^{\circ}-\beta )}{I_{\mathrm{IN}}(p,-q,\;90{}^{\circ}-\beta )}\right].$$

After substituting (34) into (35) and applying the light transmission though the polarizer *P* = *I*_p_/*I*_IN_, which remains constant for a fixed orientation of the polarizer, we obtain

(36) $$\begin{array}{ll} \bar{T}(p,q,\beta )= & \frac{1}{8}\;P\left(Q_{f}^{2}+Q_{s}^{2}\right)\left(S_{e}^{2}+S_{r}^{2}\right)\\ & +\frac{1}{4}\;\mathrm{pP}\sin\left(2\beta \right)\left(Q_{f}^{2}+Q_{s}^{2}\right)S_{e}S_{r}\cos\Gamma \\ & -\frac{1}{2}\;\mathrm{pqP}\cos\left(2\beta \right)Q_{f}Q_{s}S_{e}S_{r}\sin\gamma \sin\Gamma . \end{array}$$

By employing the approximations cos(Γ_2_*_ω_*) ≈ 1 and sin(Γ_2_*_ω_*) ≈ Γ_2*ω*_, we can express the average transmission (36) as the sum of the DC component ${\bar{T}}_{0}$ and the second harmonic component ${\bar{T}}_{2\omega }$. Proceeding analogously as in Section 2.1, we defined a time-invariant complex transmission $\underline{{\bar{T}}_{2\omega }}$ representing the amplitude and initial phase of ${\bar{T}}_{2\omega }$. Then, using the proportionality of the corresponding voltages and light intensities, it can be found that the Kerr constant is given by the following formula (see full derivation in Appendix A):

(37) $$\underline{K}=-i\frac{\sqrt{2}\;q}{\pi LE_{0}^{2}}\;\frac{\underline{A_{22}}B_{02}-\underline{B_{22}}A_{02}}{A_{02}^{2}/C+B_{02}^{2}C},$$

where

(38) $$A_{02}(p,\;q)=\frac{1}{2}\left[a_{02}(p,\;q)-a_{02}(p,-q)\right],$$

(39) $$B_{02}(p,\;q)=\frac{1}{2}\left[b_{02}(p,\;q)+b_{02}(p,-q)\right]\;,$$

(40) $$\underline{A_{22}}(p,\;q)=\frac{1}{2}\left[\underline{a_{22}}(p,\;q)-\underline{a_{22}}(p,-q)\right],$$

(41) $$\underline{B_{22}}(p,\;q)=\frac{1}{2}\left[\underline{b_{22}}(p,\;q)+\underline{b_{22}}(p,-q)\right],$$

and the coefficients $a_{02}$, $b_{02}$, $\underline{a_{22}}$, and $\underline{b_{22}}$ are given by Equations (23), (24), (26), and (27). The coefficient *C* in Equation (37) is the calibration factor for a given imperfect quarter-wave plate:

(42) $$C=\frac{2Q_{f}Q_{s}\sin\gamma }{Q_{f}^{2}+Q_{s}^{2}}=\frac{1-{\left(\Delta Q/Q\right)}^{2}}{1+{\left(\Delta Q/Q\right)}^{2}}\sin\gamma ,$$

where $\Delta Q=\left(Q_{f}-Q_{s}\right)/2$ and $Q=\left(Q_{f}+Q_{s}\right)/2$. It follows from Equation (42) that the value *C* = 1 only corresponds to an ideal quarter-wave plate, while any imperfections in terms of phase difference and transmission coefficients always lead to *C* < 1. Given that the incorrectness of phase difference in quarter-wave plates reaches values up to 10°, it can be roughly estimated that *C* ranges from 0.985 to 1.000 (neglecting dichroism).

### 2.3. Experimental Material

To test the proposed measurement method, we selected Lotos Synthetic C2+C3 SAE 5W-30 engine oil, manufactured by Lotos Oil Ltd., Gdańsk, Poland. This oil is intended for use in modern passenger cars equipped with diesel engines with particulate filters [20]. The motivation for choosing such a complex mixture was the content of long molecules that can freely perform rotational oscillations in an alternating electric field, resulting in a strong frequency dependence of the Kerr constant. Previous data have shown that this is not a common phenomenon in liquids, probably due to the formation of molecular associations that dampen the rotation (e.g., the weak effect of frequency on the Kerr constant in methyl silicone oils [5] and transformer oil [21]), or due to the very short time constant of rotational oscillations in the case of simple small molecules [22]. Moreover, the observed wide changes in the phase of the complex Kerr constant with frequency and temperature do not seem possible in a single-component liquid.

The proposed measurement method can be applied to any other liquid in which the light transmission is sufficient to ensure the correct operation of the photodetector. In addition, the electrical conductivity of the liquid must be low enough to avoid a rapid increase in temperature when the modulating voltage is switched on. In the case of well-conducting liquids, it is only possible to perform measurements using short DC voltage pulses. However, this well-known method does not take into account the changes in light transmission caused by electro-absorption, and its generalization goes beyond the scope of this work.

## 3. Experiment

**Figure 1:**
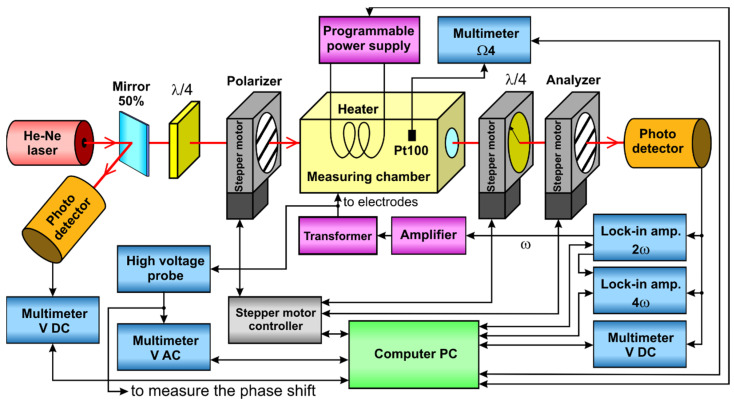
Schematic diagram of the measurement system.

The oil was poured into a 50-mm glass cuvette with stainless steel electrodes with a length *L* = 49.13 mm and Teflon spacers ensuring a gap between the electrodes of *d* = 2.78 mm. Electro-optical measurements were performed in the system shown in Figure 1. A Lasos LGK 7665 P He-Ne laser with a wavelength of 632.8 nm was used as the light source. The laser beam was split into two beams by a semi-transparent mirror. The reflected beam directly illuminated a Thorlabs PDA36A-EC photodetector connected to a DC voltmeter (Keythley 2000 multimeter), which was used to read the *U*_IN_ voltage. The beam emerging from the mirror was directed through an electro-optical modulator onto the second photodetector, a Thorlabs PDA100A-EC. The voltage from the output of this photodetector was decomposed into a constant component *U*_0_ measured by a DC voltmeter (Keythley 2000) and the components at the second and fourth harmonics of the modulating signal measured by two EG&G 7265 lock-in amplifiers. The fourth harmonic readings were not used in our calculations, but the results confirmed that higher order effects and products of terms changing with frequency 2*ω* could be neglected in the derivations presented in Section 2. One of the lock-in amplifiers was also used to generate a modulating sine-wave signal, which was amplified by a Yamaha A-S501 amplifier and a Telto TSZ 90 VA high-voltage transformer. To measure the voltage at the transformer output, an AC voltmeter (Keythley 2000) with a Tektronix P6015A high-voltage probe 1000:1 was used.

The electro-optical modulator consisted of a polarizer, an oil sample between the electrodes, a quarter-wave plate, and an analyzer. Prior to the electro-optical measurements, the parameters of a Melles Griot quarter-wave plate, model 02WRQ001/632.8, were measured to obtain *γ* = 84.2693° and |Δ*Q*|/*Q* = 0.0157, resulting in a calibration factor (42) of *C* = 0.9945. The cuvette with the oil sample and electrodes was placed in a measuring chamber designed to stabilize the oil temperature with a maximum error of ±0.2 °C. To control their azimuths, the polarizer, quarter-wave plate, and analyzer were mounted on three Thorlabs NR360S/M nanorotator stages connected to a Thorlabs BSC203 three-channel controller.

Measurements were performed for oil temperatures ranging from 25 °C to 80 °C, increased in steps of 5 °C, and for a modulating signal with frequencies of 37, 67, 87, 127, 167, 217, 317, 417, 517, 617, 717, 817, 917, and 1017 Hz. For all temperatures and frequency combinations, the control program set 18 combinations of the azimuths of the polarizer, quarter-wave plate, and analyzer. These included all 16 combinations of *α* = ±45°, *θ* = ±45°, *β* = 0°, 45°, 90°, 135° intended for measurements of the Kerr constant. In the other two combinations, there was no interference. The transmission of an extraordinary wave in the sample was observed at *α* = *θ* = *β* = 0° and the transmission of an ordinary wave at *α* = *θ* = *β* = 90°. After each change of azimuths, measurements were made for 14 voltage levels at the output of the modulating generator, which corresponded to the voltage at the electrodes increasing from approximately 715 to 2050 V RMS. All readings were repeated 15 times for each level of the modulating voltage and the results were averaged. To apply formulas (33) and (37) to readings taken at various voltage levels, we used the least squares method to fit the slope coefficient $\underline{a}$ in the relations $\left(\underline{a_{22}}b_{02}-\underline{b_{22}}a_{02}\right)/\left(a_{02}^{2}+b_{02}^{2}\right)=\underline{a}E_{0}^{2}$ and $\left(\underline{A_{22}}B_{02}-\underline{B_{22}}A_{02}\right)/\left(A_{02}^{2}/C+B_{02}^{2}C\right)=\underline{a}E_{0}^{2}$ for Formulas (33) and (37), respectively.

Because our analysis included the phase shift arising in the sample between the modulation of transmitted light and the square of the modulating voltage, it was necessary to determine the phase characteristics of the electrical circuits used. The main source of the phase shift (up to several dozen degrees) was a circuit consisting of the amplifier and the high-voltage transformer loaded with a capacitive sample. To obtain the phase characteristics of this circuit on the frequency and the sample temperature, we connected a lock-in amplifier to a high-voltage probe, bypassing the electro-optical light modulator and photodetector (see Figure 1, connector “to measure the phase shift”). Knowing the phase *ψ* of the modulating signal, we were able to apply a phase correction of −2*ψ* to the readings of the second harmonic at the photodetector output. The values obtained in this way represent the phase shift introduced by the sample together with the photodetector, its built-in amplifier, and the cables connecting the photodetector with the lock-in amplifier. To estimate the contribution of the electrical circuit itself, we repeated the measurements using an ADP (NH_4_H_2_PO_4_) crystal with painted electrodes instead of the oil sample. These measurements included both the phase of the modulating signal with the transformer loaded by the crystal and the response of the light modulator using the quadratic electro-optic effect in the crystal at the second harmonic of the modulating signal. Since the quadratic electro-optic effect in solid crystals is very fast and the mechanical resonance of the crystal was suppressed by flooding it with viscous methyl silicone oil, we considered the crystal as a standard for zero phase shift between the field-induced birefringence and the square of the applied field. Therefore, we interpreted the obtained phase shift at the second harmonic (up to several degrees depending on the frequency) as coming solely from the electrical circuits and used it to correct the phase of the second harmonic at the photodetector output obtained during the measurements of the oil sample.

The traditional approach to electro-optical measurements in a Sénarmont-type system with an alternating modulating field involves determining the modulation depth of the transmitted light for one particular operating point on the *I* on Γ characteristic given by Equation (2) such as the maximum-linearity point or the minimum-transmission point [14]. Although we did not perform this type of measurement, we can simulate it based on the data obtained for several *β* azimuth settings and expansions into series (20) and (21). Hence, the complex modulation depth at the second harmonic for a given *β* is

(43) $$\underline{m_{2\omega }}=\frac{\;\underline{U_{2\omega }}\;}{U_{0}}=\frac{\underline{a_{20}}+\underline{a_{22}}\cos2\beta +\underline{b_{22}}\sin2\beta }{a_{00}+a_{02}\cos2\beta +b_{02}\sin2\beta }.$$

Considering the maximum-linearity point on the rising slope of the *I* on Γ characteristic, we obtained 2*β* = *q*Γ_DC_ for *p* = −*q* or 2*β* = *q*Γ_DC_ + *π* for *p* = *q*, where Γ_DC_ is given by Formulas (30) and (31), while in the case of a falling slope, the conditions should be reversed. Taking into account all cases, we obtain

(44) $$\underline{m_{2\omega }}=\frac{\;\underline{U_{2\omega }}\;}{U_{0}}=\frac{1}{a_{00}}\left(\underline{a_{20}}\mp q\frac{\underline{a_{22}}b_{02}-\underline{b_{22}}a_{02}}{\sqrt{a_{02}^{2}+b_{02}^{2}}}\right)$$

and the complex Kerr constant was found assuming an ideal quarter-wave plate, *S*_e_ = *S*_r_ and *A*_e_ = *A*_r_ = 0

(45) $$\underline{K}=\pm i\frac{\sqrt{2}}{\pi LE_{0}^{2}}\underline{m_{2\omega }},$$

where the upper signs in the symbols “∓” and “±” refer to the rising edge and the lower signs refer to the falling edge.

## 4. Results and Discussion

The measurements made for 16 combinations of azimuths *α* = ±45°, *θ* = ±45°, *β* = 0°, 45°, 90°, and 135° allowed us to calculate two values of the complex Kerr constant $\underline{K}$ according to Formula (37), corresponding to different polarizer settings *α* = −45° and +45° for a given temperature and frequency. In the case of the simplified model described in Section 2.1, we obtained four values calculated from Formula (33), corresponding to four combinations of *α* = ±45° and *θ* = ±45°. Furthermore, the simulation of measurements at the maximum-linearity operating points described in Section 3 resulted in eight values calculated from Formula (45), which corresponded to all combinations of *α* = ±45°, *θ* = ±45° as well as the rising and falling edges. For each complex value of $\underline{K}$ obtained, we calculated the absolute value $K=|\underline{K}|$ and the argument φK=atan2⁡(Im⁡K_, Re⁡K_). Because a single standard deviation of 2, 4, or 8 values is rather unreliable, we averaged the results over all 168 combinations of temperature and frequency. We additionally calculated the averages over the entire temperature range from the results obtained only at the lowest frequency (37 Hz), at which the scatter of results was the largest. In the case of *φ*_K_, the sample standard deviations *s*(*φ*_K_) corresponding to various temperatures and frequencies were averaged directly. However, the values of *K* and *s*(*K*) varied by more than an order of magnitude over the entire temperature and frequency range, and so we had to average the coefficient of variation, defined as the ratio of the sample standard deviation to the sample mean $\mathrm{CV}(K)=s(K)/\bar{K}$.

As shown in Table 1, the improvement achieved using the model described in Section 2.2 over the two simpler models was so significant that we cannot explain it solely by introducing a calibration factor for an imperfect quarter-wave plate or by doubling the amount of data processed to calculate a single $\underline{K}$ value. Therefore, the improvement was mainly due to the use of Formula (35), which eliminates the terms proportional to $\left(Q_{f}^{2}-Q_{s}^{2}\right)$ and $\left(S_{e}^{2}-S_{r}^{2}\right)$ from the light transmission (34), instead of ignoring them as in both simpler models.

It is worth noting that all three measurement models considered above in Table 1 led to similar values of |*K*| when we considered the averaged results for all measurement configurations above-mentioned, and the discrepancies between the three measurement models did not exceed 2 % for any temperature and frequency. For example, for 25 °C and 37 Hz, we obtained average results of 3.42 × 10^−15^, 3.41 × 10^−15^ and 3.40 × 10^−15^ m V^−2^, respectively, for the models leading to Equations (37), (33), and (45). However, if we separately consider the measurements made in the individual configurations, it turns out that the classical measurement method and Equation (45) resulted in a particularly large scatter of results from |*K*| = 3.08 × 10^−15^ to 3.80 × 10^−15^ m V^−2^. In the case of the measurements presented here, the average value was significantly better than the individual results, but this cannot be proven for any other liquid without making additional assumptions.

**Figure 2:**
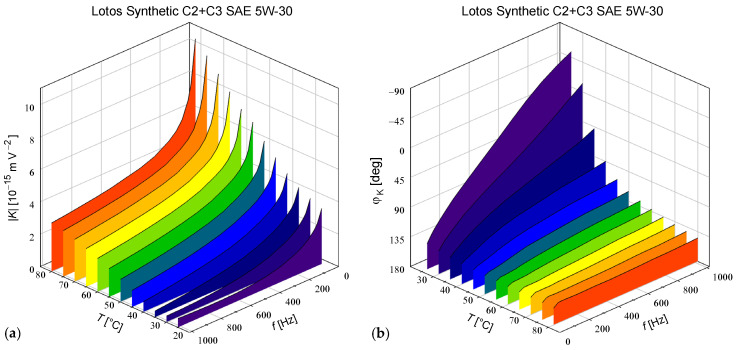
The absolute value (**a**) and argument (**b**) of the complex Kerr constant measured in Lotos Synthetic C2+C3 SAE 5W-30 oil as a function of the modulating field frequency and temperature. To increase the readability of Figure 2b, the directions of all axes *φ*_K_, *f*, and *T* are reversed compared to the analogous axes in Figure 2a.

The temperature and frequency dependences of the Kerr constant calculated using the most accurate Formula (37) and averaged over both measurement series *α* = −45° and +45° are shown in Figure 2. The obtained frequency dependences of the absolute value of the Kerr constant (Figure 2a) clearly decreased at each temperature and tended to non-zero values at high frequencies. The theoretical dependence derived in Ref. [13] using the forced rotational diffusion model also decreased, but at high frequencies decreased to zero as the expression $K\;\sim a_{1}/\omega +a_{2}/\omega^{2}$. In our opinion, this discrepancy was a result of the *K*(*t*) ~ <*P*_2_(cos *ϑ*)> approximation used in the theoretical model (where *P*_2_ is the Legendre polynominal of order 2), which means that the field-induced birefringence is a function only of the orientation of the dipoles *ϑ*, and the changes in birefringence must tend to zero as the rotational oscillations of the molecules disappear at high frequencies. Contrary to the theoretical predictions, our experimental data suggest that we also observed a contribution from electron cloud deformations, which is a relatively weak but very rapid phenomenon.

The plot in Figure 2a also shows the increasing temperature dependence of the Kerr constant *K* obtained at all frequencies. To our knowledge, this type of temperature dependence has not been reported for any other liquid, except in our previously published measurements for methyl silicone oils [5]. Moreover, there is currently no known theory that can describe the observed increasing dependence. The classical statistical-mechanical theory of the electro-optical Kerr effect by Buckingham and Raab only applies to a DC electric field and predicts a decreasing temperature dependence of the molecular Kerr constant [11], which has been experimentally confirmed for many liquids measured with a DC or pulsed field [11,23,24,25]. However, we cannot use this theory correctly when the observed induced birefringence is strongly phase shifted relative to *E*^2^, as is visible in Figure 2b. In the case of the forced rotational diffusion model, the formulas derived in [13] contain three parameters whose values must be assumed arbitrarily and their temperature dependencies are unknown. We can only suppose that the key effect is the decreasing temperature dependence of the time constant *τ*_D_ resulting from the decomposition of molecular associations and the decreasing viscosity of the liquid with increasing temperature.

Our experimental data also showed that the argument *φ*_K_ of the complex Kerr constant decreased with increasing frequency at each temperature (Figure 2b). It is worth noting that at the lowest temperature of 25 °C, we observed a great change of about −158° with increasing frequency, and the frequency dependence of *φ*_K_ gradually disappeared with increasing temperature, ending with a change of only −10° at a temperature of 80 °C. The theoretical frequency dependence of *φ*_K_ is not given directly in the literature, but it can be easily obtained from the time-dependent real Kerr constant derived in [13], which decomposes into components proportional to cos(2*ωt*) and sin(2*ωt*) for an alternating electric field with frequency *ω*. Hence, we found that in the case of a single-component liquid consisting only of permanent dipole moments, *φ*_K_ decreases monotonically with increasing frequency from 0° to −180° (for positive *K* in Equation (1) with a DC field) or from +180° to 0° (for negative DC *K*). If the liquid also contains induced dipoles, the frequency dependence of *φ*_K_ ceases to be monotonic and decreases from 0° at *ω* = 0 to a local minimum in the range (−180°…−90°), then increases to −90° for *ω* → ∞ or decreases from +180° to the local minimum (+0°…+90°), then increases to +90°. Comparison of our experimental data with these theoretical predictions suggests that we did not observe a significant contribution of induced dipole moments in the tested frequency band. Moreover, the wide range of *φ*_K_ changes with frequency changes observed at the lowest temperatures of 25 °C and 30 °C cannot be explained for a single-component liquid. In the case of engine oil, which is a mixture of many components, there are probably components with both signs of the DC Kerr constant (1) and various time constants of their rotational oscillations. Considering as a first approximation the additivity of the contributions made by individual components, this allows us to obtain any value of *φ*_K_.

**Figure 3:**
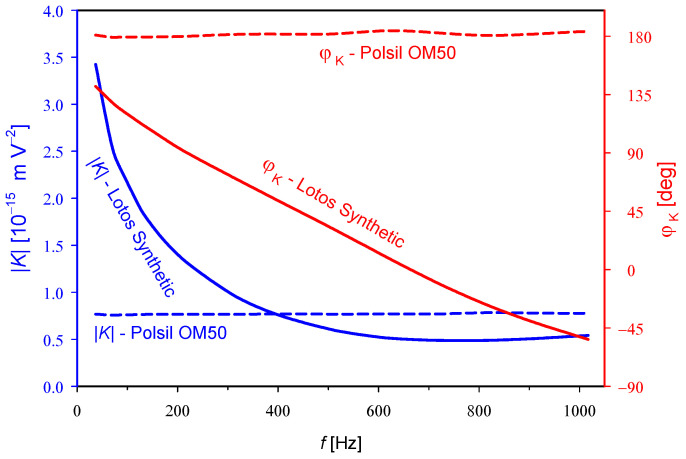
Comparison of the absolute values |*K*| and arguments *φ*_K_ of the complex Kerr constant in Lotos Synthetic C2+C3 SAE 5W-30 engine oil (solid lines) and Polsil OM50 methyl silicone oil (dashed lines) at 25 °C.

To exclude errors in the measurement procedure as the source of the observed strong frequency dependence of *φ*_K_, we performed additional measurements at a temperature of 25 °C for Polsil OM50 methyl silicone oil (viscosity 50 cSt), manufactured by Silikony Polskie Ltd., Poland [26], using the same equipment and measurement procedure that was used previously with engine oil. As can be seen in Figure 3, the methyl silicone oil showed an almost frequency-independent absolute value for the Kerr constant of 0.77 × 10^−15^ m V^−2^ and a phase close to 180° in the entire frequency band from 37 to 1017 Hz. Due to the high viscosity of this oil, the length of the molecules (a chain of about 50 dimethylsiloxane segments), and the lack of a permanent dipole moment, we can be certain that the frequency is not too low to observe a phase shift between the rotational oscillations of the molecules and the square of the applied alternating field. Therefore, the results show that there is no rotational movement of the molecules. More data on the Kerr constant in methyl silicone oils of various viscosities can be found in [5], but the phase characteristics have not been previously analyzed.

**Figure 4:**
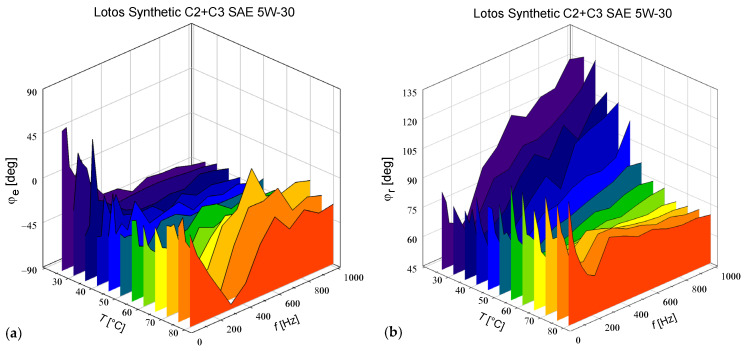
Phases *φ*_e_ (**a**) and *φ*_r_ (**b**) of the complex transmission modulation coefficients of extraordinary and ordinary waves, respectively.

The additional measurements performed in the configurations *α* = *θ* = *β* = 0° and *α* = *θ* = *β* = 90° allowed us to examine the transmission of an extraordinary wave and an ordinary wave, respectively, without their interference. As can be seen in Figure 4a, the modulation of the transmission of the extraordinary wave at the second harmonic can be shifted in phase relative to *E*^2^ by the value *φ*_e_, which strongly depends on the frequency and temperature. In the case of an ordinary wave, we observed another dependence and there was no simple correlation between any of the three phases *φ*_e_ (Figure 4a), *φ*_r_ (Figure 4b), and *φ*_K_ (Figure 2b). Consequently, a reliable separation of the contributions made by the electro-optical Kerr effect and electro-absorption cannot be based on the simple addition and subtraction of the *U*_2ω_ readings treated as real values. The phases corresponding to the *U*_2ω_ readings taken for different configurations of the modulator must also be taken into account.

## 5. Conclusions

Measurements of the Kerr constant performed in an alternating electric field provide much more information than classic measurements with a DC or pulsed field and, as shown previously, may be useful for assessing the quality and monitoring the aging processes of various industrially important liquids. Further development of this research, however, requires more general methods for measuring the Kerr constant, which will no longer apply the common assumption that the transmission coefficients of the ordinary and extraordinary waves in a liquid sample are equal and do not change because of the applied electric field. Although this assumption is well-justified for transparent liquids, many industrially important liquids including mineral, synthetic, and vegetable oils are only partially transparent in visible light. Therefore, we proposed an improved dynamic polarimetric measurement method that allows for decoupling of the electro-optical Kerr effect from changes in the transmission coefficients of both waves in a sample placed in an alternating field. The method works correctly even when the modulation of the transmission coefficients, the induced birefringence related to the Kerr effect, and the square of the modulating field do not show any phase correlation. It uses a Sénarmont-type configuration, in which readings are taken at multiple operating points on the modulator’s transmission characteristics *I*(Γ). The relative positions of these points can be controlled easily and precisely using stepper motors, and there is no need to control the absolute position of any specific operating point, as is required in the traditional approach. The more accurate of the two proposed versions of the method also includes compensation for the dichroism of the quarter-wave plate and the deviation of its actual phase difference from the ideal value of 90°.

To test the proposed method, we measured the frequency and temperature dependence of the Kerr constant in Lotos Synthetic C2+C3 SAE 5W-30 engine oil. The obtained frequency dependences decreased at each temperature and tended toward non-zero values at high frequencies. This result is partially inconsistent with the theoretical dependence derived in [13], which is also decreasing but tends to zero for *ω* → ∞. In our opinion, this discrepancy results from the fact that the theoretical model neglects the contribution of electron cloud deformations. In the low frequency range, the obtained frequency dependence of the Kerr constant cannot be directly fitted to the theoretical dependence for a single-component liquid. However, the experimental dependence seems to be consistent with the theory when we consider the oil as a sum of various components described by a negative and positive Kerr constant for *ω* → 0.

The obtained temperature dependences of the absolute value of the Kerr constant increased at each frequency of the modulating field. This tendency is opposite to the decreasing temperature dependence predicted by the classical statistical-mechanical theory of Buckingham and Raab [11]. However, their theory was developed for a DC electric field, and to our knowledge, there is no alternative theory that can correctly describe our experimental results.

The experimental results presented in this paper were obtained for the wavelength of 632.8 nm. The values of the Kerr constant for other wavelengths may be slightly different due to the theoretically predicted dependence appearing for wavelengths close to those corresponding to absorption bands [27]. However, the dispersion of the Kerr constant has still not been sufficiently investigated experimentally. The measurement method proposed in this work seems suitable for conducting reliable measurements of this dependence in future works.

If the phase shifts introduced by the electrical circuits of the measurement system are compensated, as in this study, it is also possible to measure the phase shift *φ*_K_ between the field-induced birefringence and the square of the applied alternating electric field. The wide range of observed changes in the *φ*_K_ value as a function of frequency and sample temperature means that the Kerr constant measured in an alternating field cannot be treated as a real value with a positive or negative sign, as it has been traditionally. A correct description of the effect is possible by, for example, using a time-dependent real function, as in [13]. However, treating the Kerr constant as a time-invariant complex phasor, as we have in this paper, allows for the equations to be written in a more concise form.

## Figures and Tables

**Table 1:** The coefficient of variation *CV*(*K*) for the absolute value of the Kerr constant and standard deviation *s*(*φ*_K_) for the argument of the complex Kerr constant averaged over all combinations of sample temperature and modulating field frequency or over temperature only for data obtained at 37 Hz.

**Measurement Model**	**Average *CV*(*K*) [-]**		**Average *s*(*φ*_K_) [deg]**	
	**All Frequencies**	**at 37 Hz**	**All Frequencies**	**at 37 Hz**
Section 2.2, Equation (37)	0.0056	0.011	0.14	0.26
Section 2.1, Equation (33)	0.044	0.050	0.31	0.63
Section 3, Equation (45)	0.045	0.063	2.22	6.47

## Data Availability

The raw/processed data supporting the conclusions of this article will be made available by the authors on request.

## Appendix A. Derivation of Equation (37)

Let us rewrite Equation (36) with the total phase difference Γ decomposed into the sum of the DC component Γ_DC_ and the second harmonic component Γ_2*ω*_

(A1) $$\begin{array}{ll} \bar{T}(p,q,\beta ) & =\frac{1}{8}\;P\left(Q_{f}^{2}+Q_{s}^{2}\right)\left(S_{e}^{2}+S_{r}^{2}\right)\\ & +\frac{1}{4\;}\;\mathrm{pP}S_{e}S_{r}\left[\left(Q_{f}^{2}+Q_{s}^{2}\right)\sin\left(2\beta \right)\cos\Gamma_{\mathrm{DC}}-2qQ_{f}Q_{s}\sin\gamma \cos\left(2\beta \right)\sin\Gamma_{\mathrm{DC}}\right]\cos\Gamma_{2\omega }\\ & -\frac{1}{4}\;\mathrm{pP}S_{e}S_{r}\left[\left(Q_{f}^{2}+Q_{s}^{2}\right)\sin\left(2\beta \right)\sin\Gamma_{\mathrm{DC}}+2qQ_{f}Q_{s}\sin\gamma \cos\left(2\beta \right)\cos\Gamma_{\mathrm{DC}}\right]\sin\Gamma_{2\omega }. \end{array}$$

Unlike the case of an ideal quarter-wave plate (Section 2.1), now the minimum of the DC component of the average light transmission $\bar{T}$ does not coincide with the extinction of the Kerr effect contribution to the second harmonic component. Therefore, we cannot simplify Equation (A1) using a single difference $\left(2\beta -q\Gamma_{\mathrm{DC}}\right)$, but it is possible with two differences $(2\beta -q\Gamma_{\mathrm{DC}}^{\prime })$ and $(2\beta -q\Gamma_{\mathrm{DC}}^{\prime \prime })$:

(A2) $$\begin{array}{ll} \bar{T}(p,q,\beta ) & =\frac{1}{8}\;P\left(Q_{f}^{2}+Q_{s}^{2}\right)\left(S_{e}^{2}+S_{r}^{2}\right)\\ & +\frac{1}{2}\;\mathrm{pP}S_{e}S_{r}\left[G^{\prime }\sin\left(2\beta -q\Gamma_{\mathrm{DC}}^{\prime }\right)\cos\Gamma_{2\omega }-{\mathrm{qG}}^{\prime \prime }\cos\left(2\beta -q\Gamma_{\mathrm{DC}}^{\prime \prime }\right)\sin\Gamma_{2\omega }\right], \end{array}$$

where

(A3) $$G^{\prime }=\frac{1}{2}\sqrt{{\left(Q_{f}^{2}+Q_{s}^{2}\right)}^{2}\cos^{2}\Gamma_{\mathrm{DC}}+4Q_{f}^{2}Q_{s}^{2}\sin^{2}\gamma \sin^{2}\Gamma_{\mathrm{DC}}},$$

(A4) $$G^{\prime \prime }=\frac{1}{2}\sqrt{{\left(Q_{f}^{2}+Q_{s}^{2}\right)}^{2}\sin^{2}\Gamma_{\mathrm{DC}}+4Q_{f}^{2}Q_{s}^{2}\sin^{2}\gamma \cos^{2}\Gamma_{\mathrm{DC}}},$$

(A5) $$\sin\Gamma_{\mathrm{DC}}^{\prime }=\frac{Q_{f}Q_{s}\sin\gamma }{G^{\prime }}\sin\Gamma_{\mathrm{DC}},$$

(A6) $$\cos\Gamma_{\mathrm{DC}}^{\prime }=\frac{Q_{f}^{2}+Q_{s}^{2}}{2G^{\prime }}\cos\Gamma_{\mathrm{DC}},$$

(A7) $$\sin\Gamma_{\mathrm{DC}}^{\prime \prime }=\frac{Q_{f}^{2}+Q_{s}^{2}}{2G^{\prime \prime }}\sin\Gamma_{\mathrm{DC}},$$

(A8) $$\cos\Gamma_{\mathrm{DC}}^{\prime \prime }=\frac{Q_{f}Q_{s}\sin\gamma }{G^{\prime \prime }}\cos\Gamma_{\mathrm{DC}}.$$

After substituting Equations (6)–(8) into (A2), applying the approximations cos(Γ_2_*_ω_*) ≈ 1 and sin(Γ_2_*_ω_*) ≈ Γ_2*ω*_, and neglecting all products of small terms at frequency 2*ω*, we can express $\bar{T}$ as the sum of the DC component ${\bar{T}}_{0}$ and the second harmonic component ${\bar{T}}_{2\omega }$:

(A9) $${\bar{T}}_{0}(p,q,\beta )=\frac{1}{8}\;P\left(Q_{f}^{2}+Q_{s}^{2}\right)\left(S_{e0}^{2}+S_{r0}^{2}\right)+\frac{1}{2}\;\mathrm{pP}S_{e0}S_{r0}G^{\prime }\sin\left(2\beta -q\Gamma_{\mathrm{DC}}^{\prime }\right)$$

and

(A10) $$\begin{array}{ll} {\bar{T}}_{2\omega }(p,q,\beta ) & =\frac{1}{4}P\left(Q_{f}^{2}+Q_{s}^{2}\right)\left[S_{e0}A_{e}\cos\left(2\omega t+\varphi_{e}\right)+S_{r0}A_{r}\cos\left(2\omega t+\varphi_{r}\right)\right]E_{0}^{2}\\ & +\frac{1}{2}\;\mathrm{pqP}S_{e0}S_{r0}G^{\prime \prime }\cos\left(2\beta -q\Gamma_{\mathrm{DC}}^{\prime \prime }\right)\pi L\left|K\right|E_{0}^{2}\cos\left(2\omega t+\varphi_{K}\right)\\ & +\frac{1}{2}\;\mathrm{pP}\left[S_{e0}A_{r}\cos\left(2\omega t+\varphi_{r}\right)+S_{r0}A_{e}\cos\left(2\omega t+\varphi_{e}\right)\right]E_{0}^{2}G^{\prime }\sin\left(2\beta -q\Gamma_{\mathrm{DC}}^{\prime }\right). \end{array}$$

Equation (A10) can be written in a simpler form using the complex coefficients (15)–(17) and the time-independent complex average transmission $\underline{{\bar{T}}_{2\omega }}$, which represents the amplitude and phase of the real ${\bar{T}}_{2\omega }$. Following the convention used for lock-in amplifiers in Equations (11) and (12), we assumed here that Re⁡[T¯2ω_] represents the component of ${\bar{T}}_{2\omega }$ varying as sin(2*ωt*), and Im⁡[T¯2ω_] corresponds to the component varying as cos(2*ωt*), which leads to:

(A11) $$\begin{array}{ll} \underline{{\bar{T}}_{2\omega }}(p,q,\beta )= & \mathrm{iP}\left[\frac{1}{4}\left(Q_{f}^{2}+Q_{s}^{2}\right)\left(S_{e0}\underline{A_{e}}+S_{r0}\underline{A_{r}}\right)\right.\\ & +\frac{1}{2}\;\mathrm{pq}S_{e0}S_{r0}G^{\prime \prime }\cos\left(2\beta -q\Gamma_{\mathrm{DC}}^{\prime \prime }\right)\pi L\underline{K}\\ & \left.+\frac{1}{2}\;p\left(S_{e0}\underline{A_{r}}+S_{r0}\underline{A_{e}}\right)G^{\prime }\sin\left(2\beta -q\Gamma_{\mathrm{DC}}^{\prime }\right)\right]E_{0}^{2}. \end{array}$$

As can be seen from Equation (A11), the modulation due to the Kerr effect can be decoupled from the electro-absorption contribution by calculating the following difference:

(A12) $$\begin{array}{ll} \underline{{\bar{T}}_{2\omega }}(p,q, & \beta =q\Gamma_{\mathrm{DC}}^{\prime }/2)-\underline{{\bar{T}}_{2\omega }}\left(p,\;q,\;\beta =q\Gamma_{\mathrm{DC}}^{\prime }/2+\pi /2\right)\\ & =\mathrm{ipqP}S_{e0}S_{r0}G^{\prime \prime }\cos\left(\Gamma_{\mathrm{DC}}^{\prime }-\Gamma_{\mathrm{DC}}^{\prime \prime }\right)\pi L\underline{K}E_{0}^{2}, \end{array}$$

where the value of *PS*_e0_ *S*_r0_ can be found as the doubled amplitude of the ${\bar{T}}_{0}$ on *β* dependence given by Equation (A9):

(A13) $${\bar{T}}_{0,\max}-{\bar{T}}_{0,\min}=PS_{e0}S_{r0}G^{\prime }.$$

Analogously to the definition of the average light transmission (35), we can also define the average voltage ratio from the two photodetectors and decompose it into the DC component and the complex RMS voltage measured at the second harmonic:

(A14) $${\bar{u}}_{0}(p,q,\beta )\overset{\mathrm{def}}{=}\frac{1}{2}\;\left[\frac{U_{0}(p,q,\beta )}{U_{\mathrm{IN}}(p,q,\beta )}+\frac{U_{0}(p,-q,\;90{}^{\circ}-\beta )}{U_{\mathrm{IN}}(p,-q,\;90{}^{\circ}-\beta )}\right],$$

(A15) $$\underline{{\bar{u}}_{2\omega }}(p,q,\beta )\overset{\mathrm{def}}{=}\frac{1}{2}\;\left[\frac{\underline{U_{2\omega }}(p,q,\beta )}{U_{\mathrm{IN}}(p,q,\beta )}+\frac{\underline{U_{2\omega }}(p,-q,\;90{}^{\circ}-\beta )}{U_{\mathrm{IN}}(p,-q,\;90{}^{\circ}-\beta )}\right].$$

Since the dependencies of $U_{0}/U_{\mathrm{IN}}$ and $\underline{U_{2\omega }}/U_{\mathrm{IN}}$ on *β* are described by the series (20) and (21), the average voltage ratios (A14) and (A15) can also be expressed in the form

(A16) $${\bar{u}}_{0}=A_{00}+A_{02}\cos2\beta +B_{02}\sin2\beta ,$$

(A17) $$\underline{{\bar{u}}_{2\omega }}=\underline{A_{20}}+\underline{A_{22}}\cos2\beta +\underline{B_{22}}\sin2\beta ,$$

where the coefficients $A_{00}$ and $\underline{A_{20}}$ are given by

(A18) $$A_{00}(p,\;q)=\frac{1}{2}\left[a_{00}(p,\;q)+a_{00}(p,-q)\right],$$

(A19) $$\underline{A_{20}}(p,\;q)=\frac{1}{2}\left[\underline{a_{20}}(p,\;q)+\underline{a_{20}}(p,-q)\right],$$

and the remaining coefficients by Formulas (38)–(41).

Using the proportionality *U* ~ *I*, we can write

(A20) $$\sqrt{2}\frac{\underline{{\bar{u}}_{2\omega }}\left(\beta =q\Gamma_{\mathrm{DC}}^{\prime }/2\right)-\underline{{\bar{u}}_{2\omega }}\left(\beta =q\Gamma_{\mathrm{DC}}^{\prime }/2+\pi /2\right)}{{\bar{T}}_{2\omega }\left(\beta =q\Gamma_{\mathrm{DC}}^{\prime }/2\right)-{\bar{T}}_{2\omega }\left(\beta =q\Gamma_{\mathrm{DC}}^{\prime }/2+\pi /2\right)}=\frac{{\bar{u}}_{0,\max}-{\bar{u}}_{0,\min}}{{\bar{T}}_{0,\max}-{\bar{T}}_{0,\min}}.$$

Hence, after substituting Equations (A12), (A13), (A16), and (A17), we obtain

(A21) $$\sqrt{2}\frac{2\left(\underline{A_{22}}\cos\Gamma_{\mathrm{DC}}^{\prime }+q\underline{B_{22}}\sin\Gamma_{\mathrm{DC}}^{\prime }\right)}{\mathrm{ipqP}S_{e0}S_{r0}G^{\prime \prime }\cos\left(\Gamma_{\mathrm{DC}}^{\prime }-\Gamma_{\mathrm{DC}}^{\prime \prime }\right)\pi L\underline{K}E_{0}^{2}}=\frac{2\sqrt{A_{02}^{2}+B_{02}^{2}}}{PS_{e0}S_{r0}G^{\prime }},$$

where $\sin\Gamma_{\mathrm{DC}}^{\prime }$ and $\cos\Gamma_{\mathrm{DC}}^{\prime }$ result from the proportionality of analogous terms containing sin 2*β* and cos 2*β* in Equations (A9) and (A16):

(A22) $$\cos\Gamma_{\mathrm{DC}}^{\prime }=\frac{pB_{02}}{\sqrt{A_{02}^{2}+B_{02}^{2}}},$$

(A23) $$\sin\Gamma_{\mathrm{DC}}^{\prime }=\frac{-\mathrm{pq}A_{02}}{\sqrt{A_{02}^{2}+B_{02}^{2}}}.$$

Using Equations (A5)–(A8), (A22), and (A23), it can be shown that

(A24) $$\begin{array}{c} \frac{G^{\prime }}{G^{\prime \prime }\cos\left(\Gamma_{\mathrm{DC}}^{\prime }-\Gamma_{\mathrm{DC}}^{\prime \prime }\right)}=\frac{G^{\prime }}{G^{\prime \prime }\left(\cos\Gamma_{\mathrm{DC}}^{\prime }\cos\Gamma_{\mathrm{DC}}^{\prime \prime }+\sin\Gamma_{\mathrm{DC}}^{\prime }\sin\Gamma_{\mathrm{DC}}^{\prime \prime }\right)}\\ =\frac{1}{\cos^{2}\Gamma_{\mathrm{DC}}^{\prime }\frac{2Q_{f}Q_{s}\sin\gamma }{Q_{f}^{2}+Q_{s}^{2}}+\sin^{2}\Gamma_{\mathrm{DC}}^{\prime }\frac{Q_{f}^{2}+Q_{s}^{2}}{2Q_{f}Q_{s}\sin\gamma }}\\ =\frac{A_{02}^{2}+B_{02}^{2}}{B_{02}^{2}\frac{2Q_{f}Q_{s}\sin\gamma }{Q_{f}^{2}+Q_{s}^{2}}+A_{02}^{2}\frac{Q_{f}^{2}+Q_{s}^{2}}{2Q_{f}Q_{s}\sin\gamma }}. \end{array}$$

After substituting Equations (A22)–(A24) into (A21), we finally obtain the constant $\underline{K}$ given by Formula (37).
